# Impact of inhibitory KIR ligand mismatch and other variables on outcomes following myeloablative posttransplant cyclophosphamide-based T-cell-replete haploidentical bone marrow transplantation

**DOI:** 10.3389/fimmu.2024.1413927

**Published:** 2024-12-16

**Authors:** Sarah Kayser, Emilia Salzmann-Manrique, Hubert Serve, Peter Bader, Jan-Henning Klusmann, Christian Seidl, Joachim Schwäble, Gesine Bug, Evelyn Ullrich

**Affiliations:** ^1^ Department of Medicine 2, University Hospital, Goethe University Frankfurt, Frankfurt, Germany; ^2^ Department of Hematology, Oncology and Cancer Immunology, Charité - Universitätsmedizin Berlin, corporate member of Freie Universität Berlin and Humboldt-Universität zu Berlin, Berlin, Germany; ^3^ German Cancer Consortium (DKTK), Partner site Frankfurt/Mainz, a partnership between DKFZ and University Medical Center Frankfurt, Frankfurt, Germany; ^4^ Department of Pediatrics, Goethe University Frankfurt, Frankfurt, Germany; ^5^ Frankfurt Cancer Institute, Goethe University, Frankfurt, Germany; ^6^ Institute for Transfusion Medicine and Immunohematology, German Red Cross Blood Donor Service, Goethe University Hospital Medical School, Frankfurt, Germany

**Keywords:** hematopoietic cell transplantation (HCT), T-cell-repleted haploidentical bone marrow transplantation (BMT), posttransplant cyclophosphamide (PTCy), hematological malignancies, inhibitory KIR ligand mismatch, recipient and donor variables, immune reconstitution

## Abstract

**Introduction:**

Posttransplant cyclophosphamide (PTCy) has revolutionized the landscape of human leukocyte antigen (HLA)-haploidentical hematopoietic cell transplantation (haplo-HCT), providing a pivotal therapeutic option for patients with hematological malignancies who lack an HLA-matched donor.

**Methods:**

In this retrospective analysis involving 54 adult patients undergoing PTCy-based haplo-HCT, we evaluated the impact of inhibitory killer immunoglobulin-like receptor (KIR)/HLA mismatch, alongside patient, donor, and transplant factors, on clinical outcomes within a homogeneous cohort characterized by a myeloablative conditioning regimen and bone marrow graft.

**Results:**

With a median follow-up of 73.2 months, our findings reveal promising outcomes: 6-year overall survival, relapse-free survival, and graft-versus-host disease (GVHD) and relapse-free survival rates were 63% (95% CI: 51–79), 58% (95% CI: 46–74), and 42% (95% CI: 30–58), respectively. Notably, the cumulative incidence rates of relapse and non-relapse mortality at 6 years post-haplo-HCT were 29% (95% CI: 19–45) and 12% (95% CI: 6–26), respectively. Acute GVHD at day 100 posttransplantation occurred with a cumulative incidence of 33% (95% CI: 22– 49) for grades II–IV and 9% (95% CI: 3–23) for grades III–IV. Furthermore, 41% of patients developed chronic GVHD within 1 year posttransplantation, distributed as follows: 28% mild, 9% moderate, and 4% severe.

**Conclusion:**

Within our cohort, several variables were associated with outcomes following PTCy-based haplo-HCT. However, inhibitory KIR/HLA mismatch did not influence these outcomes.

## Highlights

- This study underscores the potential of PTCy-based haplo-HCT as a curative option for hematological malignancies.
- Inhibitory KIR/HLA mismatch did not significantly affect any of the key HCT outcomes.

## 1 Introduction

Posttransplant cyclophosphamide (PTCy) has emerged as a paradigm-shifting strategy in allogeneic hematopoietic cell transplantation (allo-HCT), particularly in the context of human leukocyte antigen (HLA)-haploidentical HCT (haplo-HCT) (1). Haplo-HCT represents a curative treatment option for patients with hematological malignancies and disorders who lack a fully HLA-matched donor (2). Despite this advancement, a significant proportion of patients undergoing PTCy-based haplo-HCT experience disease recurrence or non-relapse mortality (NRM). NRM is most commonly attributed to complications such as graft-versus-host disease (GVHD) and infections. GVHD is characterized by alloreactive T-cell recognition of host antigens leading to immune-mediated tissue damage (3). Infections are due to immunosuppression and delayed immune reconstitution after allo-HCT. This underscores the critical need to comprehensively understand the factors influencing transplant outcomes to optimize patient survival. Individual patient characteristics and disease features undeniably influence the success of haplo-HCT; however, they are often not modifiable. Increasingly, attention has turned toward donor-related factors, given the expanding pool of potential donors available for haplo-HCT. As patients may have multiple potential haploidentical donors, selecting the most suitable donor has become pivotal. Donor selection criteria traditionally include considerations such as HLA matching, donor age, gender, and cytomegalovirus (CMV) status, alongside other factors such as ABO compatibility and overall health. Additionally, recent studies have highlighted the potential significance of inhibitory killer immunoglobulin-like receptor (KIR)/HLA mismatch between the donor and recipient in influencing transplant outcomes (4–11). KIRs are receptors expressed on natural killer (NK) cells that recognize HLA molecules, thus modulating NK cell activity. The principal ligands for inhibitory KIRs are found within the HLA-C group, categorized into C group 1 and C group 2 based on polymorphism at residue 80 in the HLA-C molecule or Bw4 epitopes. Upon binding of an inhibitory KIR to its ligand, the NK cell is inhibited. Infected and malignant cells frequently downregulate HLA expression, inducing NK cell-mediated killing. When the host lacks a ligand present in the donor, donor NK cells may become activated against host cells. Notably, KIR ligand mismatching in the GVH direction has been linked with lower relapse rates, improved engraftment, and reduced rates of GVHD in T-cell-depleted haplo-HCT (12, 13). However, the impact of inhibitory KIR/HLA mismatch on outcomes following PTCy-based haplo-HCT remains a subject of debate. Prior studies have yielded conflicting results, potentially due to the heterogeneity in patient cohorts, conditioning regimens, and graft sources utilized (4–11). In this retrospective single-center study, we address these gaps by evaluating the influence of inhibitory KIR/HLA mismatch alongside patient, donor, and transplant characteristics on clinical outcomes in a homogeneous cohort of 54 patients undergoing PTCy-based haplo-HCT using a myeloablative conditioning regimen and bone marrow (BM) graft.

## 2 Methods

### 2.1 Study design and patient population

We included all adult patients with any hematologic malignancy who underwent myeloablative PTCy-based haploidentical bone marrow transplantation (haplo-BMT) between September 2011 and December 2019 at our institution. The study was conducted in accordance with the ethical standards of the institutional and national research committee (ethic vote # UCT-2-2019) and with the 1964 Helsinki Declaration and its later amendments or comparable ethical standards. All patients provided informed consent for the use of their medical record data for research purposes.

### 2.2 PTCy-based haplo-BMT

For patients diagnosed with myeloid disorders, the conditioning regimen consisted of either thiotepa (5 mg/kg on days −6 and −5), busulfan (3.2 mg/kg on days −4 to −2), and fludarabine (50 mg/m^2^ on days −4 to −2) (TBF) or fludarabine (30 mg/m^2^ on days −5 to −2), melphalan (140 mg/m^2^ on day −11), and fractionated total body irradiation (TBI, 2–8 Gy on days −1 to 0) (FMT). Patients with lymphoid diseases received a conditioning regimen comprising fractionated TBI (2 × 2 Gy on days −8 to −6) and fludarabine (30 mg/m^2^ on days −5 to −2) (FTBI). For elderly patients (>55 years), the busulfan was shortened to either 1 or 2 days and TBI was reduced to 2 days. On day 0, T-cell-replete BM with a target dose of 4 × 10^8^/kg mononuclear cells was administered. Patients receiving peripheral blood stem cells (PBSCs) were excluded from the study. Posttransplant, patients were given PTCy at a dose of 50 mg/kg on days +3 and +5. Cyclosporine A was initiated on day 0, while mycophenolate mofetil was started on day +1. A subset of patients received tacrolimus due to a change in local practice. For patients without GVHD, mycophenolate mofetil was discontinued between days +35 and +42, and cyclosporine A/tacrolimus was discontinued between days +120 and +180. Pegylated granulocyte-colony stimulating factor 6 mg was administered on day +6 to support hematopoietic reconstitution. The patients received anti-infective prophylaxis according to local standards.

### 2.3 HLA and KIR genotyping

High-resolution typing for the HLA-A, B, C, and DPB1 loci was performed for donor and recipient samples by sequence-based typing using the AlleleSEQR kit (GenDX). Typing of the KIR genes was carried out by the polymerase chain reaction-based sequence-specific primer method (Olerup SSP). KIR ligands (C1, C2, and Bw4) were determined based on HLA class I typing. Inhibitory KIR mismatches were defined by the presence of inhibitory KIR receptors in the donor and the lack of the respective ligand in the recipient as described before (14). KIR genotypes were determined based on the presence or absence of activating KIR: the KIR AA genotype was defined by the presence of only KIR2DS4 as an activating KIR gene and a KIR B+ genotype by the presence of several activating KIR genes (15). HLA-DPB1 mismatches were defined based on T-cell-epitope (TCE) groups and classified into mismatches that might be tolerable (permissive) and those that would increase risk (non-permissive) after unrelated donor HCT (16).

### 2.4 Evaluation of immune reconstitution

Immune reconstitution was evaluated in the first year after PTCy-based haplo-HCT. The distribution and activation status of immune subpopulations were quantified via flow cytometry from patients’ whole blood. The percentages of lymphocytes, CD4^+^ T cells, CD8^+^ T cells, CD19^+^ B cells, CD56^+^CD16^+^ NK cells, and activated T cells (CD8^+^CD38^+^ and CD3^+^HLA-DR^+^ T cells) were determined using the FACS Canto II flow cytometer and the FACS Canto software (BD Biosciences, Germany). Absolute cell counts of immune cell subtypes were calculated based on the lymphocyte count by the Sysmex analysis.

### 2.5 Variables

The following patient-, disease-, donor-, and transplant-related variables were analyzed: patient variables included recipient gender, age, and hematopoietic cell transplantation-specific comorbidity index (HCT-CI) (17). Disease-related variables comprised diagnosis and disease risk index (DRI) (18). Donor variables encompassed relationship, gender, age, CMV status, inhibitory KIR/HLA mismatch, KIR genotype, and HLA-DP mismatch. Transplant-related variables included conditioning regimen and CD34^+^ and CD3^+^ cell counts in the graft.

### 2.6 Endpoints and statistical analysis

The median follow-up time was obtained by the reverse Kaplan–Meier method. Patients’ demographics and clinical characteristics were summarized as frequencies and percentages or medians and ranges, as appropriate. These characteristics were compared between donor–recipient KIR ligand groups using Pearson’s chi-square test or Wilcoxon–Mann–Whitney test. The impact of inhibitory KIR/HLA mismatch on the following endpoints was explored: neutrophil and thrombocyte engraftment, overall survival (OS), relapse-free survival (RFS), GVHD and relapse-free survival (GRFS), cumulative incidence (CI) of relapse (CIR), NRM, and CI of acute and chronic GVHD and CMV in addition to other viral reactivations/infections. All endpoints were calculated from the time of haplo-HCT. Neutrophil engraftment was defined as the first of three consecutive days with a neutrophil count >0.5 × 10^9^/L. Platelet engraftment was defined as the first of three consecutive days with a platelet count >20 × 10^9^/L in the absence of platelet transfusion for 7 consecutive days. Acute GVHD was classified as clinically significant (grades 2–4) or severe (grades 3–4) according to the modified Glucksberg criteria (19). Chronic GVHD was classified as mild, moderate, or severe according to the National Institutes of Health consensus criteria (20). OS was defined as the probability of survival regardless of disease state at any time, while RFS was defined as survival without relapse or disease progression. GRFS was defined as the time from HCT to the first occurrence of any of the following events: acute GVHD grade III–IV, chronic GVHD requiring systemic immunosuppressive therapy, disease relapse or progression, or death from any cause. Patients who did not experience any of these events were censored at the date of their last follow-up (21). Univariate analysis of OS, RFS, and GRFS was performed by the Kaplan–Meier method and tested using the log-rank test. CIR, defined as the time from transplantation to the first hematological relapse, was evaluated considering NRM as a competing risk. NRM was defined as death by any cause without prior relapse. The CIs of engraftment, acute GVHD, and chronic GVHD were analyzed considering death as a competing risk. Univariate analysis of outcomes with a competing event was performed using the Gray test. To analyze the dynamics of immune reconstitution within the patient cohort and between groups, we used a joint model for longitudinal and time-to-event data. We performed a linear mixed effect with cubic B-splines as a submodel for the longitudinal data. Cox proportional hazard for OS was considered as a submodel for time-to-event data. All tests were two-sided, *P <*0.05 was considered significant for Fisher’s exact test and Wilcoxon rank sum test, and *P <*0.10 was considered significant for every survival outcome. We provided their 6-year estimates with their corresponding 95% confidence intervals. Data analysis was performed using the R software for statistical computing, version 4.3 (R Foundation for Statistical Computing, Vienna, Austria; URL http://www.R-project.org/) and the JM package for model fitting.

## 3 Results

### 3.1 Patient, disease, donor, and transplant characteristics

Patient, disease, and transplant characteristics were well balanced between patients with and without inhibitory HLA/KIR mismatch except for a significantly higher median donor age in the inhibitory HLA/KIR mismatch group (**Table 1**). The majority of patients were men (67%) and had a median age of 56 years at the time of haplo-HCT. Acute myeloid leukemia (AML) was the predominant diagnosis (54%). Non-myeloid disorders accounted for 31% of cases. Regarding donor characteristics, the median age was 37 years, with the majority of donors being men (65%), and children constituting the most common donor type (46%). Conditioning regimens primarily included TBF (69%), followed by FTBI (20%) and FMT (11%). Cyclosporine A was used in 76% of cases and tacrolimus in 24%.

**Table 1:** Patient, disease, donor, and transplant characteristics.

	Entire cohort (*N* = 54)	No inhibitory KIR/HLA mismatch (*N* = 26)	Inhibitory KIR/HLA mismatch (*N* = 28)	*P*
Haplo-HCT, date				0.697
Median	January 2017	April 2017	November 2016	
Range	2011-09–2019-12	2011-09–2019-12	2012-04–2019-12	
Recipient gender, *N* (%)				0.700
Male	36 (67)	18 (69)	18 (64)	
Recipient age at HCT, years				0.467
Median (range)	56 (22, 75)	56 (23, 73)	56 (22, 75)	
HCT-CI, score				0.234
Mean (SD)	1.65 (1.78)	1.35 (1.44)	1.93 (2.04)	
HCT-CI, *N* (%)				0.321
Low (0)	21 (39)	11 (42)	10 (36)	
Intermediate (1–2)	16 (30)	9 (35)	7 (25)	
High (≥3)	17 (31)	6 (23)	11 (39)	
Diagnosis, *N* (%)				0.382
Myeloid	37 (69)	16 (62)	21 (75)	
AML	29 (54)	11 (42)	18 (64)	
CML	1 (2)	0 (0)	1 (4)	
MDS	7 (13)	5 (19)	2 (7)	
Non-myeloid	17 (31)	10 (38)	7 (25)	
ALL	10 (19)	6 (23)	4 (14)	
NHL	7 (13)	4 (15)	3 (11)	
DRI, *N* (%)				0.342
Low	5 (9)	2 (8)	3 (11)	
Intermediate	28 (52)	12 (46)	16 (57)	
High	14 (26)	8 (31)	6 (21)	
Very high	7 (13)	4 (15)	3 (11)	
Donor relation group, *N* (%)				0.326
Parents	8 (15)	2 (8)	6 (21)	
Sibling	17 (31)	7 (27)	10 (36)	
Children	25 (46)	15 (58)	10 (36)	
Extended family	4 (7)	2 (8)	2 (7)	
Recipient–donor gender, *N* (%)				0.351
Male–male	23 (43)	14 (54)	9 (32)	
Female–male	12 (22)	5 (19)	7 (25)	
Male–female	13 (24)	4 (15)	9 (32)	
Female–female	6 (11)	3 (12)	3 (11)	
Donor age, years				0.007
Median (range)	37 (18, 74)	33 (19, 56)	41 (18, 74)	
CMV recipient–donor, *N* (%)				0.887
Neg–Neg	9 (17)	5 (19)	4 (14)	
Neg–Pos	9 (17)	4 (15)	5 (18)	
Pos–Neg	5 (9)	3 (12)	2 (7)	
Pos–Pos	31 (57)	14 (54)	17 (61)	
Donor KIR genotype, *N* (%)				0.893
AA	15 (28)	7 (27)	8 (29)	
B+	39 (72)	19 (73)	20 (71)	
HLA-DP, *N* (%)				0.279
Non-permissive	14 (26)	5 (19)	9 (32)	
Matched/permissive	40 (74)	21 (81)	19 (68)	
Conditioning, *N* (%)				0.978
TBF	37 (69)	18 (69)	19 (68)	
FTBI	11 (20)	5 (19)	6 (21)	
FMT	6 (11)	3 (12)	3 (11)	
Immunosuppression, *N* (%)				0.637
Cyclosporine A	41 (76)	19 (73)	22 (79)	
Tacrolimus	13 (24)	7 (27)	6 (21)	
CD34^+^, 10^6^/kg				0.244
Median (range)	3.08 (1.50, 8.37)	3.08 (1.50, 8.37)	3.02 (1.57, 6.27)	
CD3^+^ T cells, 10^7^/kg				0.945
Median (range)	3.28 (1.52, 5.12)	3.35 (1.52, 5.12)	3.24 (1.56, 5.00)	

*P*-values were determined using Fisher’s exact test or Mann–Whitney *U* test.

ALL, acute lymphoblastic leukemia; AML, acute myeloid leukemia; CML, chronic myelogenous leukemia; CMV, cytomegalovirus; FMT, fludarabine, melphalan, total body irradiation; TBI, fludarabine, total body irradiation, haplo-HCT, haploidentical hematopoietic cell transplantation; HCT-CI, hematopoietic cell transplantation-specific comorbidity index; MDS, myelodysplastic syndrome; NHL, non-Hodgkin lymphoma; TBF, thiotepa, busulfan, fludarabine.

### 3.2 Neutrophil and thrombocyte engraftment

All patients achieved engraftment (**Table 2**). The CI of neutrophil engraftment at 28 days posttransplantation was high across the entire cohort, with an estimate of 93% (95% CI: 81–97). The median time to engraftment was 18 days (range 14–61) and was similar between patients with no inhibitory KIR/HLA mismatch and those with inhibitory KIR/HLA mismatch. Regarding platelet engraftment, the CI at 28 days was observed in 64.2% (95% CI: 49–75) of patients for the entire cohort, reaching 100% by day 100. The median time to platelet engraftment was 26 days (range 14–90). There was no difference between the inhibitory KIR/HLA mismatch group and the non-inhibitory KIR/HLA mismatch group.

**Table 2:** Outcomes.

	Entire cohort (*N* = 54)	No inhibitory KIR/HLA mismatch (*N* = 26)	Inhibitory KIR/HLA mismatch (*N* = 28)	*P*-value
Survival follow-up, median (range), months	73.7 (3.3–153.9)	73.7 (4.6–153.9)	76.4 (3.3–125.1)	0.688
Neutrophil engraftment				1
28-day CI, % (95% CI)	93 (81–97.1)	92 (71–98)	93 (73–98)	
Median (range) time to engraftment, days	18 (14–61)	18 (14–61)	17.5 (14–33)	
Thrombocyte engraftment				0.090
28-day CI, % (95% CI)	64.2 (49–75)	58 (34–73)	70 (47–83)	
100-day CI, % (95% CI)	100	100	100	
Median (range) time to engraftment, days	56 (14–90)	27.5 (18–90)	25 (14–45)	
Survival outcomes				
6-year OS, % (95% CI)	63 (51–79)	72 (55–95)	55 (38–78)	0.180
6-year RFS, % (95% CI)	58 (46–74)	67 (50–89)	51 (35–74)	0.180
6-year GRFS, % (95% CI)	42 (30–58)	52 (35–76)	33 (10–57)	0.109
6-year CIR, % (95% CI)	29 (19–45)	24 (12–49)	34 (20–58)	0.425
6-year NRM, % (95% CI)	12 (6–26)	9 (2–36)	15 (6–36)	0.467
Acute GVHD				
100-day CI grades II–IV, % (95% CI)	33 (22–49)	32 (18–57)	32 (18–58)	0.691
100-day CI grades III–IV, % (95% CI)	9 (3–23)	12 (4–35)	4 (1–27)	0.440
Chronic GVHD				
1-year CI, % (95% CI)	36 (25–52)	27 (15–52)	44 (29–67)	0.323
1-year CI moderate/severe, % (95% CI)	9 (4–22)	8 (2–30)	18 (8–40)	0.266
CMV reactivation				
180-day CI, % (95% CI)	74 (62–88)	67 (49–90)	79 (64–97)	0.406

*P*-values were determined using the log-rank test or Gray’s test as appropriate.

95% CI, 95% confidence interval; CI, cumulative incidence; CIR, cumulative incidence of relapse; GRFS, GVHD- and relapse-free survival; GVHD, graft-versus-host disease; HLA, human leukocyte antigen; KIR, killer cell immunoglobulin-like receptor; NRM, non-relapse mortality; OS, overall survival; RFS, relapse-free survival.

### 3.3 Follow-up

The median follow-up period extended to 73.2 months (95% CI: 61.6–92.1; range 3.3–154). Half of the patients underwent transplantation before January 2017, while the other half underwent transplantation between January 2017 and December 2019. Importantly, our analysis did not reveal any discernible era effect (**Supplementary Table S1**).

### 3.4 Outcomes of the entire cohort

**Figure 1:**
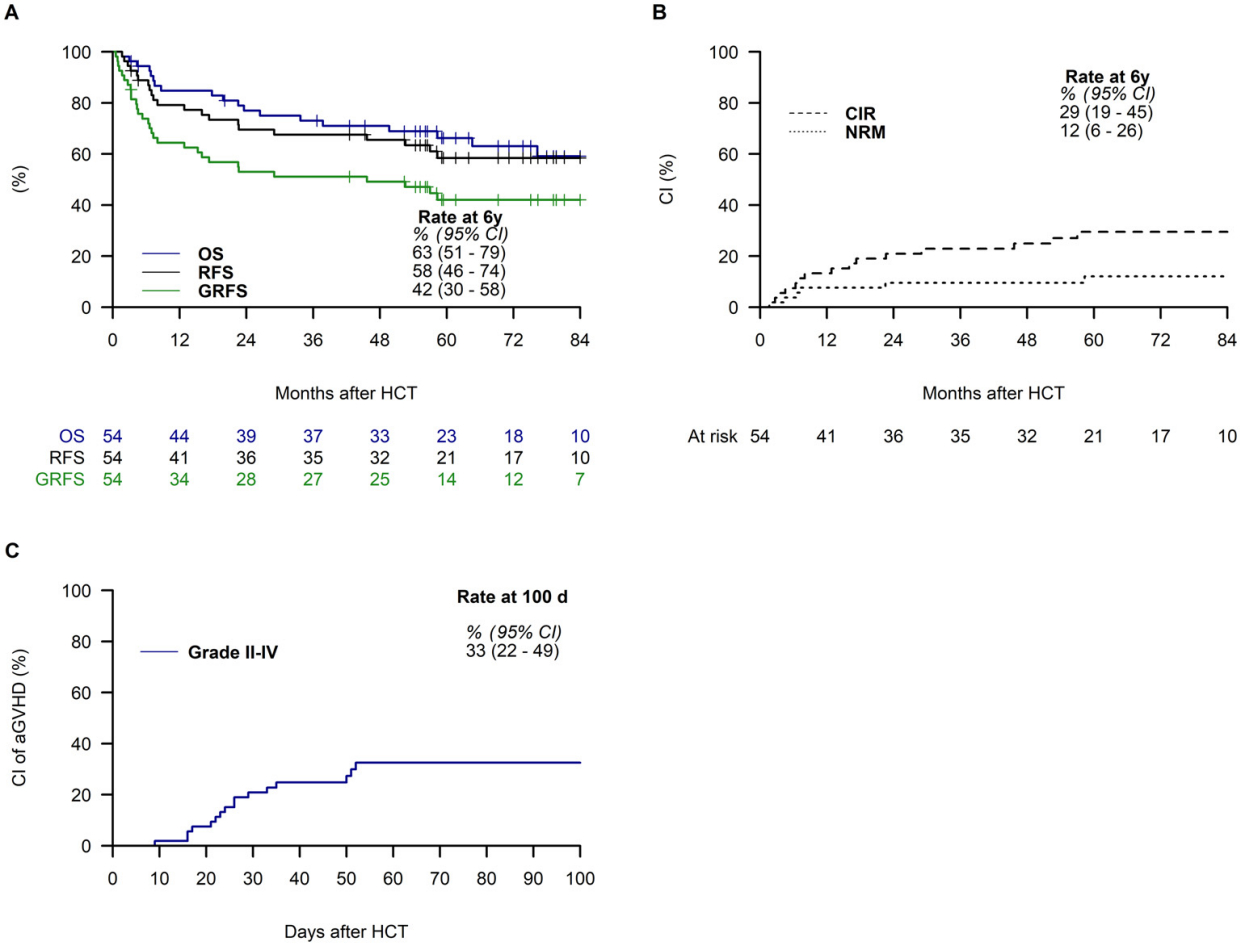
Outcomes of the entire cohort. The plots display the survival curves for overall survival (OS), relapse-free survival (RFS), and graft-versus-host disease-free, relapse-free survival (GRFS) **(A)**, cumulative incidence of relapse (CIR), and non-relapse mortality (NRM) **(B)**, and cumulative incidence (CI) for acute graft-versus-host disease grades II–IV (GVHD) **(C)**, along with estimates and a 95% confidence interval (95% CI) at 6 years posttransplantation **(A, B)** and at day 100 **(C)**.

For the entire cohort, the estimated OS at 6 years was 63% (95% CI: 51–79) (**Figure 1A**). The main cause of death was relapse (*N* = 13), followed by infection (*N* = 3) (**Supplementary Table S2**). The estimated 6-year RFS rate was 58% (95% CI: 46–74) (**Figure 1A**). Additionally, the estimated 6-year GRFS was 42% (95% CI: 30–58) (**Figure 1A**). In total, 15 patients experienced relapse, resulting in a 6-year CI rate of 29% (95% CI:19-45) (**Figure 1B**). The 6-year CI rate of NRM in the study cohort was 12% (95% CI: 6–26) (**Figure 1B**). Within the first 100 days posttransplant, the CI of acute GVHD was 33% (95% CI: 22–49) for grade II–IV acute GVHD (**Figure 1C**).

### 3.5 Outcomes based on inhibitory KIR ligand mismatch

**Figure 2:**
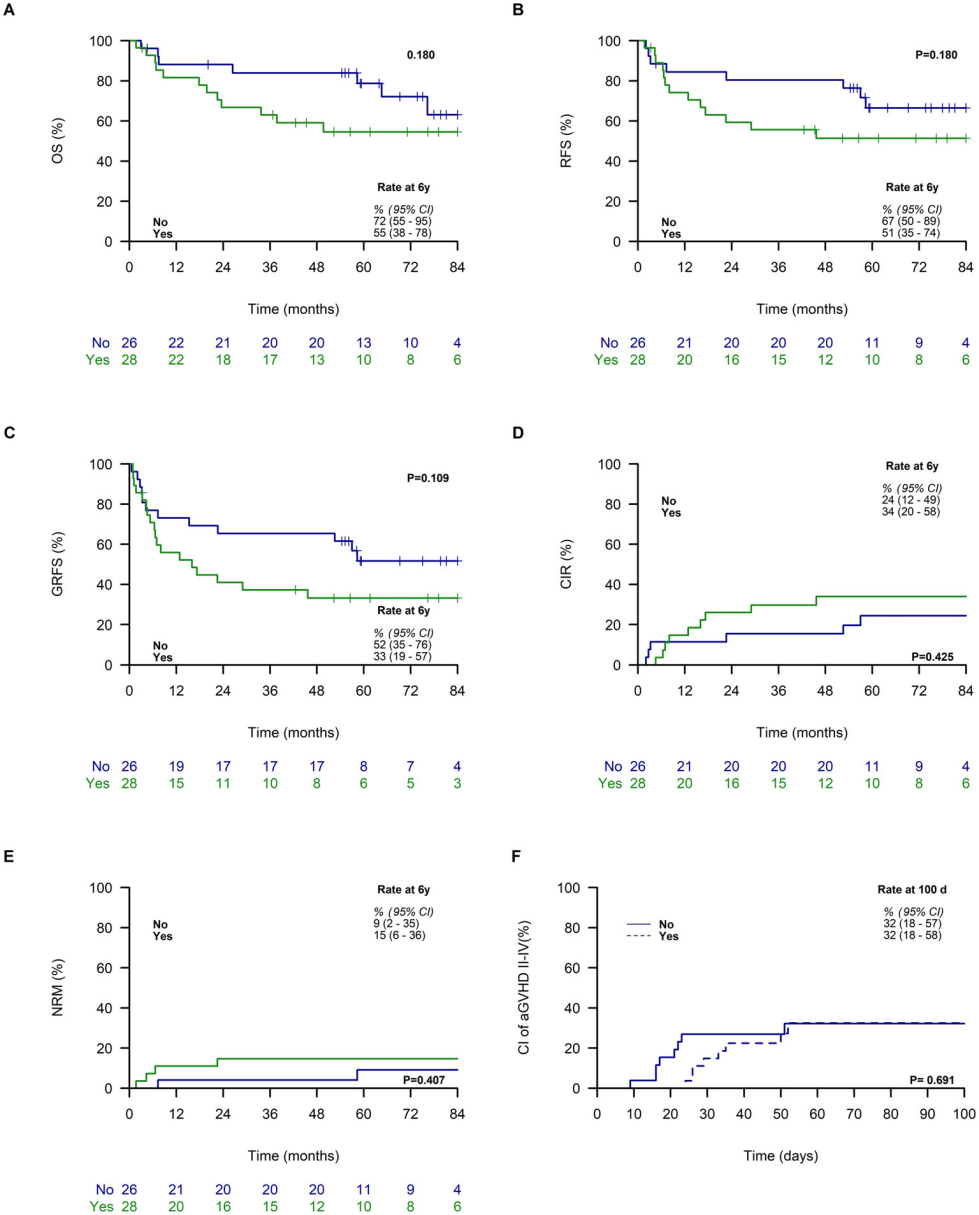
Outcomes Based on KIR Ligand Mismatch. The figures illustrate the following curves categorized by inhibitory KIR/HLA mismatch (no vs. yes): overall survival (OS) **(A)**, relapse-free survival (RFS) **(B)**, graft-versus-host disease, relapse-free survival (GFRS) **(C)**, cumulative incidence of relapse (CIR) **(D)**, and non-relapse mortality (NRM) **(E)**, and acute graft-versus-host disease grade II-IV (GVHD) **(F)**, alongside estimates and 95% confidence intervals (95% CI) at 6 years post-transplantation **(A–E)** and at day 100 **(F)**.

No significant differences were observed among patients based on inhibitory KIR/HLA mismatch status. The estimated OS at 6 years was 72% (95% CI: 55–95) for patients lacking inhibitory KIR/HLA mismatch, compared to 55% (95% CI: 38–78) for those with inhibitory KIR/HLA mismatch (**Figure 2A**). Similarly, the 6-year RFS showed no significant difference between the no inhibitory KIR/HLA mismatch group (67%, 95% CI: 50–89) and the inhibitory KIR/HLA mismatch group (51%, 95% CI: 35–74; **Figure 2B**). The 6-year GRFS was also comparable, with rates of 52% (95% CI: 35–76) in the no mismatch group and 33% (95% CI: 19–57) in the mismatch group (**Figure 2C**). Furthermore, there was no significant difference in the 6-year CIR between patients with and without inhibitory KIR/HLA mismatch (24%, 95% CI: 12–49 vs. 34%, 95% CI: 20–58; **Figure 2D**). The 6-year CI rate of NRM was also similar between the two groups (9%, 95% CI: 2–35 vs. 15%, 95% CI: 6–36; **Figure 2E**). Finally, no difference was found in the CI of acute GVHD between patients with and without inhibitory KIR/HLA mismatch (32%, 95% CI: 18–57 vs. 32%, 95% CI: 18–58; **Figure 2F**).

### 3.6 Variables associated with overall survival

**Figure 3:**
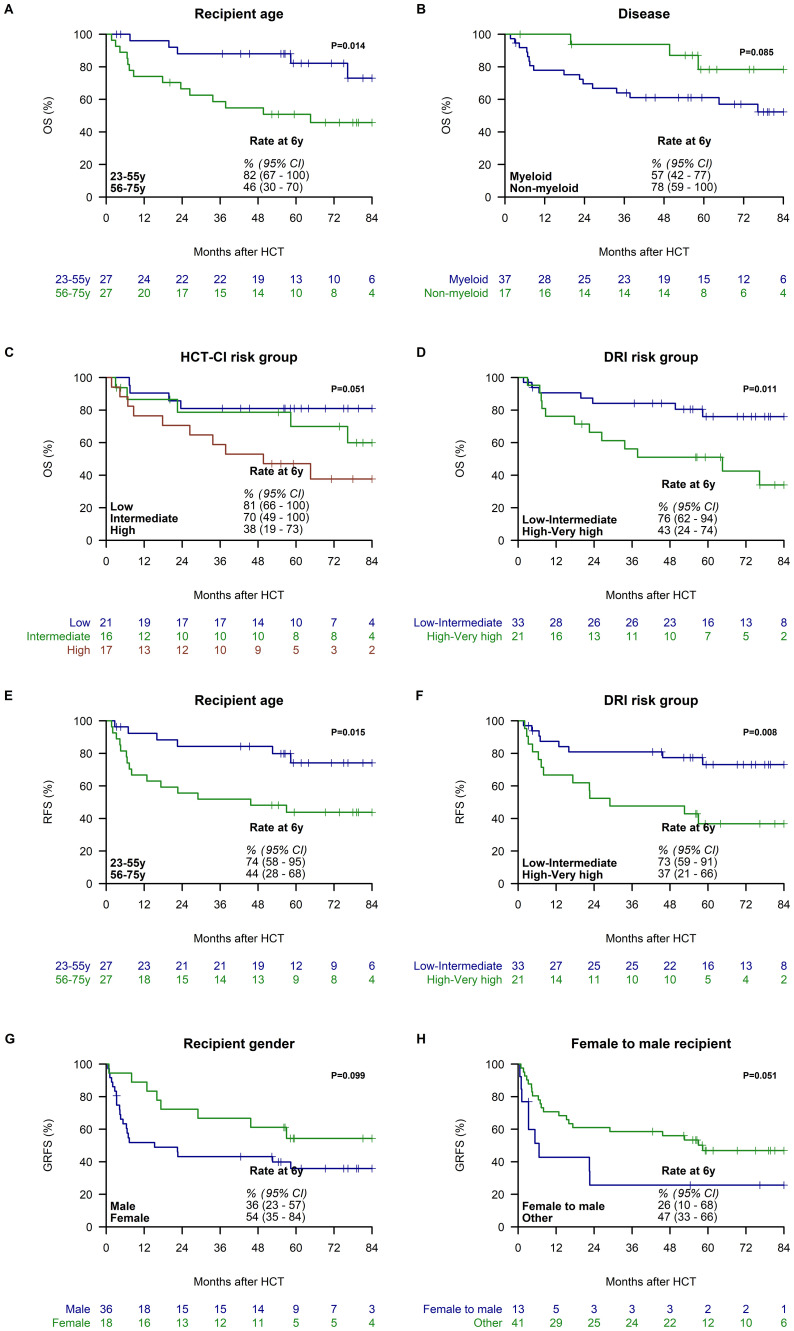
Variables Associated with Overall Survival (OS), Relapse-Free Survival (RFS) and Graft-versus-host disease-free, Relapse-free Survival (GRFS). OS is stratified by recipient age groups (23-55 vs. 56-75 years) **(A)**, disease (myeloid vs. non-myeloid) **(B)**, HCT-CI score (low vs. intermediate vs. high) **(C)**, and Disease Risk Index (DRI) risk groups (low/intermediate vs. high/very high) **(D)**. RFS is stratified by recipient age groups (23-55 vs. 56-75 years) **(E)**, and DRI risk group (low/intermediate vs. high/very high) **(F)**. GRFS is stratified by recipient gender (male vs. female) **(G)** and female donor to male recipient (no vs. yes) **(H)**. All Kaplan-Meier curves depict 6-year estimates with 95% confidence intervals (95% CI).

In univariate analysis, younger recipient age (≤55 years) at the time of haplo-HCT was associated with improved 6-year OS (82%, 95% CI: 67–100) compared to older recipients (46%, 95% CI: 30–70; **Figure 3A**). Additionally, patients transplanted for non-myeloid diseases exhibited better OS than those with myeloid disorders (78%, 95% CI: 59–100 vs. 57%, 95% CI: 42–77; **Figure 3B**). Furthermore, patients classified in the low and intermediate HCT CI categories had better survival compared to those classified as high risk (81%, 95% CI: 66–100 vs. 70%, 95% CI: 49–100 vs. 38%, 95% CI: 19–73; **Figure 3C**). Patients in the low/intermediate DRI categories showed an association with improved OS compared to those in the high/very high-risk categories (76%, 95% CI: 62–94 vs. 43%, 95% CI: 24–74; **Figure 3D**). Other patient, disease, donor, and transplant characteristics did not show an association with OS (**Supplementary Table S3**).

### 3.7 Variables associated with relapse-free survival

In univariate analyses, younger recipient age (≤55 years) at the time of haplo-HCT was found to be associated with improved 6-year RFS (74%, 95% CI: 58–95) compared to older recipients (44%, 95% CI: 28–68; **Figure 3E**). Furthermore, patients classified in the low/intermediate DRI categories demonstrated a correlation with improved RFS compared to those categorized as high/very high risk (73%, 95% CI: 59–91 vs. 37%, 95% CI: 21–66; **Figure 3F**). Other patient, disease, donor, and transplant characteristics did not demonstrate significant associations with improved RFS outcomes (**Supplementary Table S4**).

### 3.8 Variables associated with graft-versus-host disease-free, relapse-free survival

In univariate analysis, female recipients were associated with superior 6-year GRFS (54%, 95% CI: 35–84 vs. 36%, 95% CI: 23–57; **Figure 3G**), and female donor to male recipients were associated with inferior GRFS compared to other gender matches (26%, 95% CI: 10–68 vs. 47%, 95% CI: 33-66; **Figure 3H**). Other variables were not significantly associated with GRFS (**Supplementary Table S5**).

### 3.9 Variables associated with a cumulative incidence of relapse

**Figure 4:**
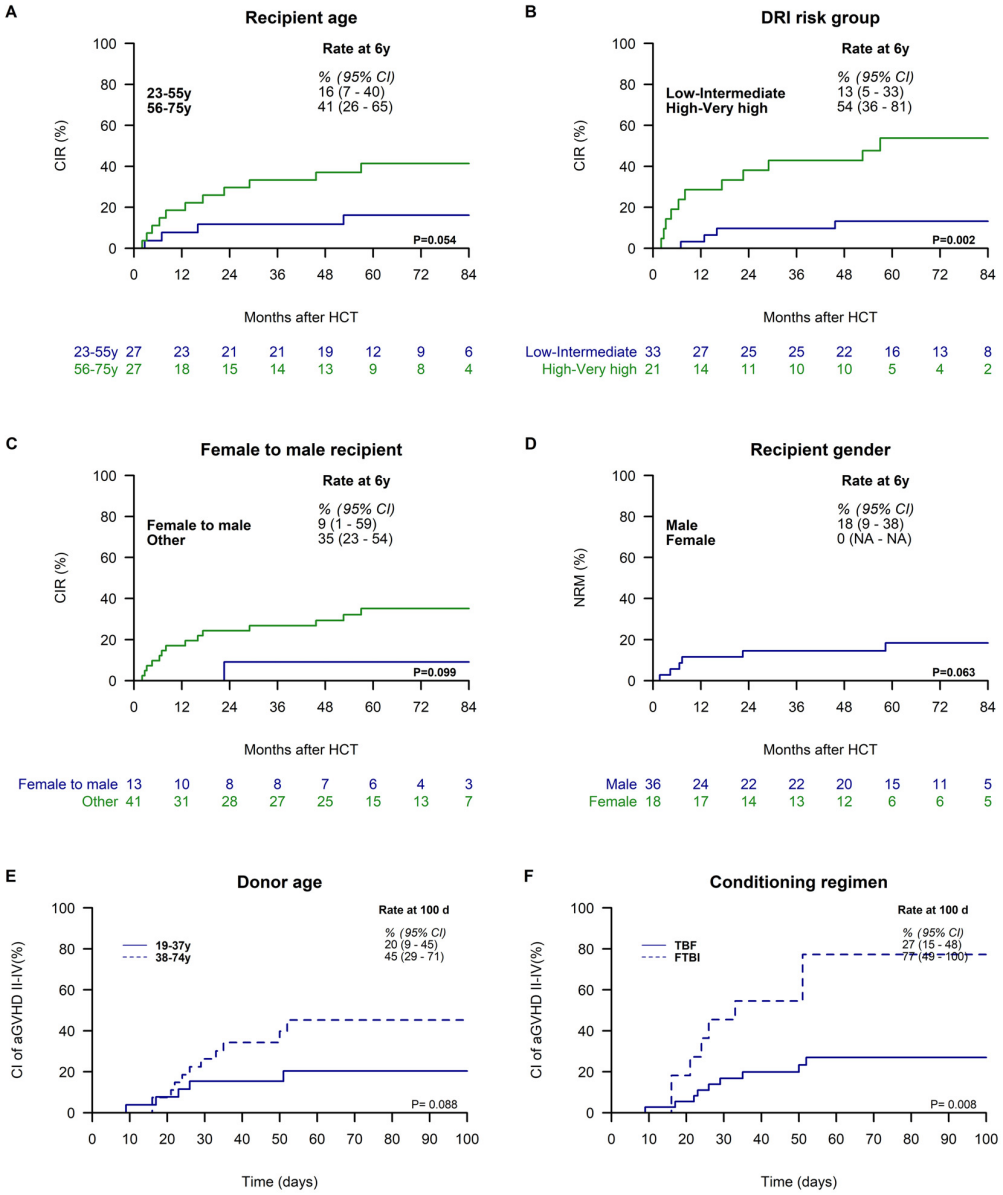
Variables Associated with Cumulative Incidence of Relapse (CIR), Non-Relapse Mortality (NRM), and Acute Graft-versus-Host Disease (GVHD) Grade II-IV.CIR is stratified by recipient age groups (23–55 vs. 56–75 years) **(A)**, Disease Risk Index (DRI) risk groups (low/intermediate vs. high/very high) **(B)**, and female donor to male recipient status (no vs. yes) **(C)**. NRM is stratified by recipient gender (male vs. female) **(D)**. The cumulative incidence of acute GVHD grade II-IV is stratified by donor age (19–37 vs. 38–74 years) **(E)** and conditioning regimen (FTBI vs. TBF or TMF) **(F)**. All Kaplan-Meier curves display 6-year estimates with 95% confidence intervals (95% CI), except for panel C, which presents estimates at day 100 post-transplantation.

In univariate analysis, younger recipient age (≤55 years) at the time of haplo-HCT was associated with a lower 6-year CIR compared to older recipients (16%, 95% CI: 7–40 vs. 41%, 95% CI: 26–65; **Figure 4A**). Similarly, patients with low/intermediate risk DRI had a lower CIR compared to those with high/very high-risk DRI (13%, 95% CI: 5–33 vs. 54%, 95% CI: 36–81; **Figure 4B**). Additionally, a female donor to a male recipient was linked to a lower CIR compared to other gender matches (9%, 95% CI: 1–59 vs. 35%, 95% CI: 23–54; **Figure 4C**). Other patient, disease, donor, and transplant characteristics did not show significant associations with CIR (**Supplementary Table S6**).

### 3.10 Variables associated with non-relapse mortality

In univariate analysis, female recipients were associated with a lower 6-year NRM compared to male recipients (0% vs. 18%, 95% CI: 9–38; **Figure 4D**). No other investigated factors were found to be statistically associated with NRM (**Supplementary Table S7**).

### 3.11 Variables associated with acute GVHD grades II–IV

In univariate analysis, younger donor age (19–37 years) was associated with a reduced CI of grade II–IV acute GVHD compared to older donor age (38–74 years) (20%, 95% CI: 9–45 vs. 45%, 95% CI: 29–71; **Figure 4E**), and conditioning with FTBI was significantly associated with an increased CI of grade II–IV acute GVHD, reaching 77% (95% CI: 49–100), in contrast to 27% (95% CI: 15–48) observed in non-FTBI regimens (**Figure 4F**). Other patient, disease, donor, and transplant characteristics did not show significant associations with grade II–IV acute GVHD (**Supplementary Table S8**).

### 3.12 CMV reactivation and other viral infections

**Figure 5:**
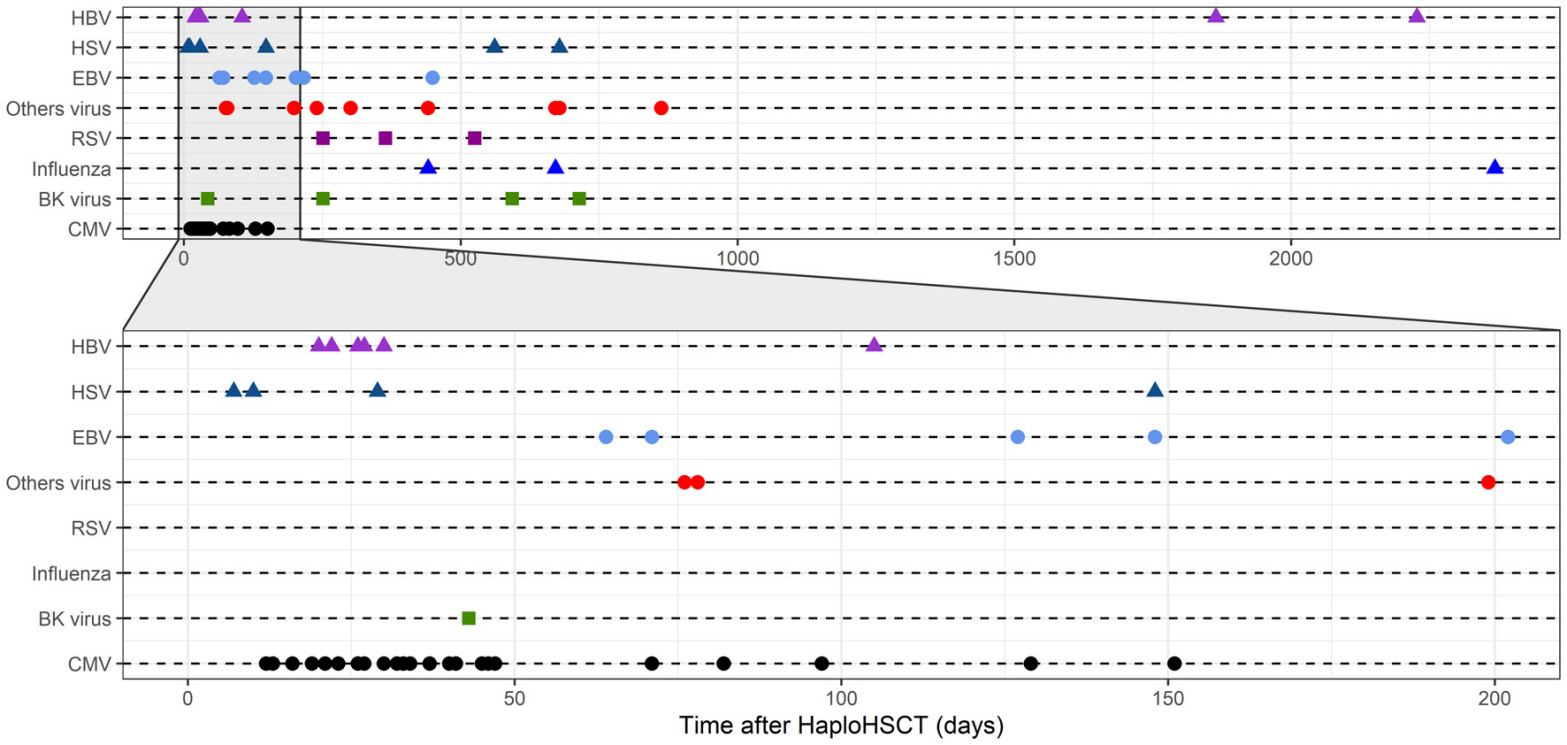
Viral reactivations and infections over time. The plot depicts the occurrence of specific viral reactivations and infections within the first 2,000 days after transplant. Each reactivation or infection is represented, with shapes and colors denoting specific viruses. The plot emphasizes the time period from 0 to 200 days after transplant, which is zoomed in to facilitate detailed examination.

Among patients considered at risk for CMV reactivation, CMV reactivated in 72% of cases, with no significant differences observed between the groups with and without inhibitory KIR/HLA mismatch (**Table 2**). In the entire cohort, 27% of patients experienced at least one viral reactivation or infection. Following CMV, the most common pathogens were respiratory viruses (31%), HHV6 (17%), EBV (12%), and HSV (11%). BK virus reactivation was observed in 7% of cases, while HBV reactivation occurred in 1 case (**Supplementary Table S9**). The majority of viral infections occurred within the first 200 days posttransplantation, with herpes viruses being predominant within the initial 50 days (**Figure 5**).

### 3.13 Immune reconstitution

**Figure 6:**
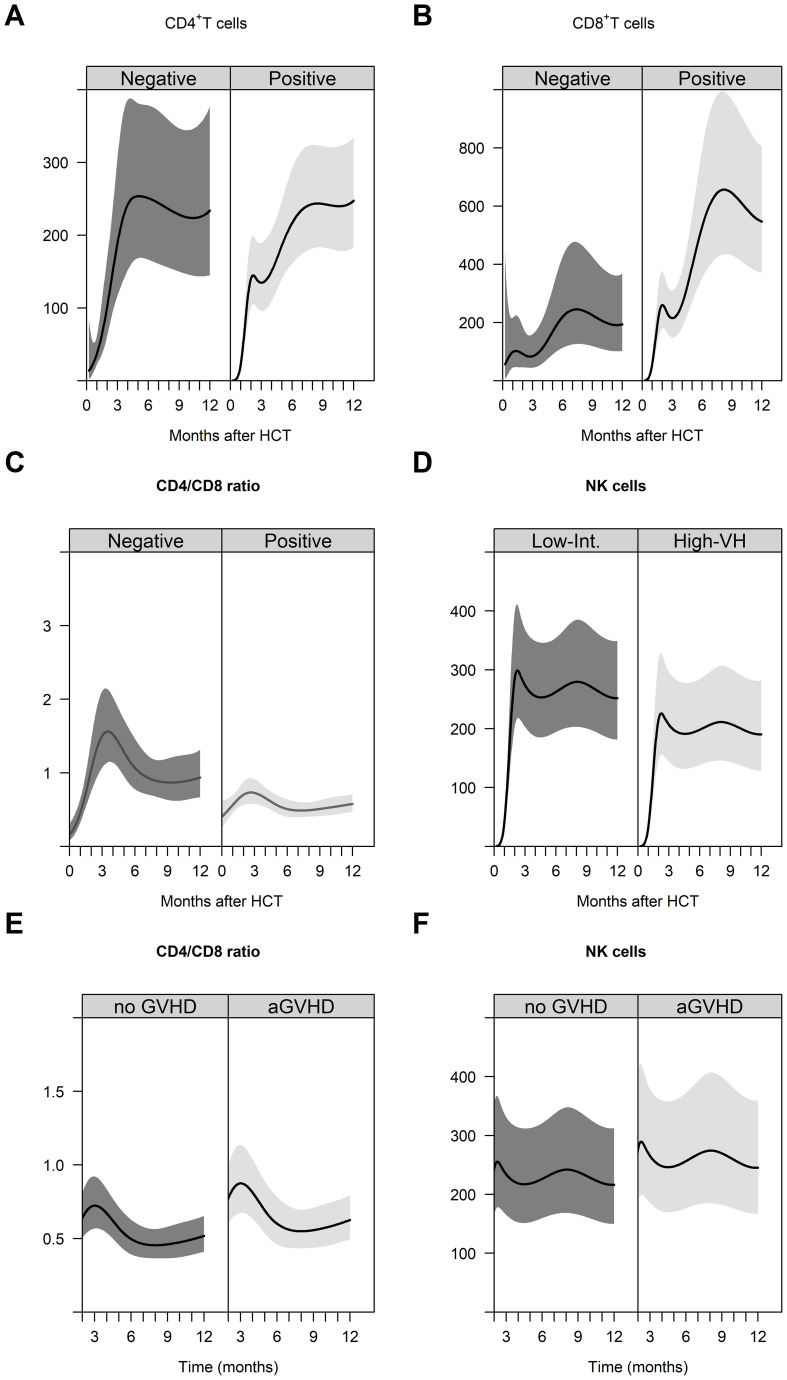
Immune reconstitution in the first year after PTCy-based haplo-HCT. Immune reconstitution of helper T cells **(A)**, cytotoxic T cells **(B)**, and CD4^+^/CD8^+^ ratio **(C)** by donor CMV status. Immune reconstitution of NK cells by DRI **(D)**. Dynamics of the CD4^+^/CD8^+^ ratio **(E)** and immune reconstitution of NK cells **(F)** from the third month (landmark at 3 months after transplantation) to the first year after transplantation according to acute GVHD.

In our study, we analyzed immune reconstitution in 53 patients over 459 days after PTCy-based haplo-HCT (**Table 3**). Leukocyte counts increased significantly from 2,496 cells/μL at month 1 to 5,801 cells/μL at month +3, remaining stable thereafter. Lymphocyte subsets showed a consistent increase post-haplo-HCT: total lymphocyte count rose from 173 cells/μL at month +1 to 1,329 cells/μL at month +12. T cells, B cells, and NK cells exhibited similar recovery patterns. Patients with CMV-positive donors showed lower CD4^+^ T-cell recovery (**Figure 6A**) but higher CD8^+^ T-cell counts (**Figure 6B**), resulting in a lower CD4/CD8 ratio (**Figure 6C**) within the first year post-allo-HCT. Notably, patients with a low/intermediate DRI had a lower NK cell recovery (**Figure 6D**). Finally, patients with acute GVHD exhibited a higher CD4/CD8 ratio (**Figure 6E**) and higher NK cell recovery (**Figure 6F**) within the first year posttransplantation.

**Table 3:** Predicted absolute values (cells/µL) from the joint model with a 95% confidence interval.

Cells	Month +1	Month +3	Month +6	Month +9	Month +12
Leukocytes	2,496	5,801	4,657	5,601	5,890
	(2,143–2,909)	(5,001–6,729)	(4,064–5,338)	(4,855–6,461)	(5,030–6,897)
Lymphocytes	173	918	1,040	1,273	1,329
	(136–219)	(735–1,146)	(852–1,270)	(1,032–1,572)	(1,054–1,676)
T cells	77	457	527	720	770
	(59–100)	(356–586)	(417–667)	(563–920)	(590–1,005)
Helper T cells	22	174	203	262	277
	(17–28)	(138–221)	(163–253)	(210–328)	(217–353)
Cytotoxic T cells	50	244	335	469	501
	(37–66)	(187–318)	(265–423)	(367–598)	(383–657)
Ratio CD4^+^/CD8^+^	0.49	0.71	0.58	0.49	0.71
	(0.40–0.60)	(0.58–0.86)	(0.48–0.70)	(0.39–0.60)	(0.42–1.20)
NK cells	36	280	230	254	232
	(27–47)	(214–366)	(174–304)	(188–342)	(172–313)
B cells	0.08	5.68	38.98	24.40	40.18
	(0.03–0.20)	(2.17–14.82)	(14.33–106.01)	(8.81–67.58)	(15.42–104.71)

NK, natural killer.

## 4 Discussion

In this retrospective study, we investigated the impact of inhibitory KIR ligand mismatch and other variables on outcomes following haplo-HCT. All patients received a uniform myeloablative conditioning regimen and bone marrow as the stem cell graft, followed by a modified PTCy protocol. The modified protocol included the administration of cyclosporine A/tacrolimus on day 0, initiation of mycophenolate mofetil on day +1, and PTCy on days +3 and +5, differing from the original protocol, which administered PTCy on days +3 and +4 with cyclosporine A and mycophenolate mofetil starting on day +5. The study had a median follow-up of 72.3 months. Our platform demonstrated promising results with acceptable OS, RFS, and GRFS, indicating effective disease control with manageable toxicity, consistent with previous studies (2, 22).

We did not observe an impact of inhibitory KIR ligand mismatch on key outcomes after haplo-HCT. This aligns with the findings by Russo et al. and others, suggesting that the benefits of inhibitory KIR ligand mismatch observed in other transplant protocols without PTCy may be less pronounced or negated in PTCy-based regimens due to blunted NK cell alloreactivity in this transplantation setting. Our study adds to the exciting literature by utilizing a uniform patient cohort with a consistent myeloablative conditioning regimen and bone marrow as the stem cell source, along with a long follow-up period in terms of outcomes. Furthermore, our data underscore that the effects of blunted NK cell alloreactivity persist even in a modified PTCy protocol, including patients transplanted under this modified approach.

However, several studies have reported an association between KIR mismatch and outcomes after PTCy (4–11), highlighting the complexity of KIR genetics and their impact on transplantation outcomes. Variations in patient cohorts, transplant platforms, NK alloreactivity prediction models, and the oversimplification of NK alloreactivity models may explain these differences. A recent study analyzing 5,017 cases of matched unrelated allo-HCT found that multiple proposed models of KIR classification did not correlate with the outcomes in multivariable analyses, suggesting limitations in using current donor KIR genotype information (23). Future studies should integrate the evolving knowledge of KIR genetics and NK cell biology to create more comprehensive models focused on homogeneous patient groups and treatment modalities.

The limitations of our study, such as its retrospective nature, modest sample size, and heterogeneity of underlying patient diseases, should be acknowledged. These limitations may have restricted our ability to detect subtle effects, emphasizing the need for larger, prospective studies in more homogeneous patient populations and treatment settings.

Regarding donor-related factors, our analysis did not identify significant influences on outcomes except for a potential association between female donors and reduced GRFS in male recipients along with a reduced relapse rate, consistent with the existing literature. In contrast, several patient-related characteristics such as older age, myeloid disease, high HCT-CI, and high/very high DRI were associated with inferior outcomes, underscoring the importance of personalized treatment strategies for these subgroups.

Additionally, while the kinetics of immune reconstitution and its impact on post-HCT outcomes are well documented in HLA-matched transplants, data remain limited for haploidentical HCT with PTCy. Our findings suggest comparable rates of viral reactivation, infections, and immune reconstitution to other allo-HCT platforms, influenced notably by CMV reactivation and GVHD.

In conclusion, our findings suggest that KIR ligand mismatch did not significantly impact outcomes after myeloablative haplo-HCT in our study cohort. Consequently, further research integrating cutting-edge knowledge on KIR genetics and NK cell biology in larger, prospective studies focusing on homogeneous patient groups and treatment modalities is needed to provide more definitive insights and enhance clinical outcomes.

## Data Availability

The patient data supporting the findings of this study are available from the corresponding author upon request. To protect patient confidentiality, all data will be provided in a blinded format and in accordance with applicable ethical and legal regulations.
