# Supplementary material for: Impact of inhibitory KIR ligand mismatch and other variables on outcomes following myeloablative posttransplant cyclophosphamide-based T-cell-replete haploidentical bone marrow transplantation
Source: Front Immunol. 2024 Dec 16;15:1413927. doi: 10.3389/fimmu.2024.1413927 (PMC11683009; doi:10.3389/fimmu.2024.1413927)
Supplement: Supplementary file 1 [file Table1.docx]

**Supplementary Material**

**Suppplementary Table 1 Outcomes by Time Period**

|  | **HCT from 2011 to Feb 2017** | **HCT from March 2017 to 2019** | **P value** |
| --- | --- | --- | --- |
| Number of patients | 27 | 27 |  |
| 6-year OS, % (95% CI %) | 65 (49 - 86) | 62 (44 - 86) | 0.814 |
| 6-year RFS, % (95% CI %) | 61 (44 - 83) | 56 (39 - 80) | 0.613 |
| 6-year GRFS, % (95% CI %) | 45 (29 - 70) | 38 (23 - 64) | 0.464 |
| 6-year CIR, % (95% CI %) | 28 (15 - 52) | 31 (17 - 55) | 0.624 |
| 6-year NRM, % (95% CI %) | 12 (4 - 33) | 14 (5 - 41) | 0.94 |
| 100-day CI acute GVHD II-IV | 29 (15 - 56) | 36 (21 - 61) | 0.468 |

Abbreviations: 95 % CI, 95 % confidence interval; CI, cumulative incidence; CIR, cumulative incidence of relapse; GRFS, GVHD- and relapse-free survival, GVHD, graft-versus-host disease; HCT, hematopoietic cell transplantation; NRM, non-relapse mortality, OS, overall survival; RFS, relapse-free survival; P values determined using log-rank test or gray test as appropriate.

**Supplementary Table 2 Cause of death**

| Cause of death | N |
| --- | --- |
| Relapse | 13 |
| Non-relapse mortality |  |
| Septic shock | 3 |
| Intracranial bleeding | 1 |
| Lung carcinoma | 1 |
| Unkown | 1 |
| Total | **19** |

**Supplementary Table 3 Variables Not Significantly Associated with Overall Survival (OS)**

|  | **N** | **6-year OS estimate (95% CI), %** | **P value** |
| --- | --- | --- | --- |
| **Recipient gender** |  |  | 0.553 |
| Male | 36 | 64 (49 - 83) |  |
| Female | 18 | 61 (40 -94) |  |
| **Donor gender** |  |  | 0.769 |
| Male | 35 | 64 (50 - 83) |  |
| Female | 19 | 61 (39 - 93) |  |
| **Female donor to male recipient** | |  | 0.467 |
| No | 41 | 60 (46 - 79) |  |
| Yes | 13 | 73 (51 - 100) |  |
| **Recipient-donor relation** |  |  | 0.039* |
| Father/mother | 8 | 71 (45 - 100) |  |
| Sister/brother | 17 | 78 (58 - 100) |  |
| Son/daughter | 25 | 58 (41 - 82) |  |
| Niece/nephew | 4 | 25 (5 - 100) |  |
| **Donor age** |  |  | 0.636 |
| 19 - 37 years | 26 | 70 (53 - 92) |  |
| 38 - 74 years | 28 | 57 (40 - 81) |  |
| **CMV recipient-donor status** |  |  | 0.114 |
| Negative-negative | 9 | 57 (29 - 100) |  |
| Negative-positive | 9 | 89 (71 - 100) |  |
| Positive-negative | 5 | 40 (14 - 100) |  |
| Positive-positive | 31 | 61 (45 - 82) |  |
| **Donor KIR genotype** |  |  | 0.491 |
| AA | 15 | 60 (40 - 91) |  |
| B+ | 39 | 64 (48 - 84) |  |
| **HLA DP** |  |  | 0.587 |
| Non permissive | 14 | 61 (39 - 95) |  |
| Matched/Permissive | 40 | 63 (49 - 82) |  |
| **Conditioning regimen** |  |  | 0.136 |
| TBF | 37 | 59 (44 - 78) |  |
| FTBI | 11 | 90 (73 - 100) |  |
| FMT | 6 | 50 (22 - 100) |  |
| **CD34^+^ cell count in the graft** |  |  | 0.934 |
| ≤ 3.08 x 10^6^/kg | 26 | 60 (43 - 83) |  |
| > 3.08 x 10^6^/kg | 25 | 62 (43 - 88) |  |
| **T cell count in the graft** |  |  | 0.258 |
| ≤ 3.28 x 10^7^/kg | 27 | 54 (38 - 77) |  |
| > 3.28 x 10^7^/kg | 26 | 72 (54 - 96) |  |
| **Immunosuppression** |  |  | 0.042** |
| Cyclosporine A | 41 | 70 (56 - 87) |  |
| Tacrolimus | 13 | 45 (24 - 83) |  |

P values determined using log-rank test.

* The result is not considered significant because the significant p-value for the OS curve in the recipient-donor relation is due to the Niece and Nephew group. Other curves show no significant differences. With only 4 patients in the Niece and Nephew group, the p-value is not robust and thus not meaningful.

** The results are not considered significant due to differences in group size and confounding variables, including recipient age, HCT-CI score, and DRI, which are associated with the outcome.

**Supplementary Table 4 Variables Not Significantly Associated with Relapse-free Survival (RFS)**

|  | **N** | **6-year RFS estimate (95% CI), %** | **P value** |
| --- | --- | --- | --- |
| **Gender recipient** |  |  | 0.654 |
| Male | 36 | 58 (43 - 78) |  |
| Female | 18 | 60 (41 - 88) |  |
| **Disease** |  |  | 0.111 |
| Myeloid | 37 | 52 (37 - 71) |  |
| Non-myeloid | 17 | 73 (54 - 100) |  |
| **HCT-CI score** |  |  | 0.116 |
| Low | 21 | 76 (60 - 97) |  |
| Intermediate | 16 | 57 (36 - 91) |  |
| High | 17 | 39 (21 - 73) |  |
| **Donor gender** |  |  | 0.45 |
| Male | 35 | 56 (42 - 76) |  |
| Female | 19 | 63 (43 - 92) |  |
| **Female donor to male recipient** | |  | 0.292 |
| No | 41 | 54 (41 - 73) |  |
| Yes | 13 | 73 (51 - 100) |  |
| **Recipient-donor relation** |  |  | 0.072* |
| Father/mother | 8 | 71 (45 - 100) |  |
| Sister/brother | 17 | 75 (56 - 100) |  |
| Son/daughter | 25 | 55 (38 - 79) |  |
| Niece/nephew | 4 | 0 (NA - NA) |  |
| **Donor age** |  |  | 0.688 |
| 19 - 37 years | 26 | 62 (44 - 86) |  |
| 38 - 74 years | 28 | 55 (39 - 78) |  |
| **CMV recipient-donor status** |  |  | 0.124 |
| Negative-negative | 9 | 53 (28 - 100) |  |
| Negative-positive | 9 | 89 (71 - 100) |  |
| Positive-negative | 5 | 20 (3 - 100) |  |
| Positive-positive | 31 | 58 (42 - 80) |  |
| **Donor KIR genotype** |  |  | 0.63 |
| AA | 15 | 53 (32 - 86) |  |
| B+ | 39 | 61 (46 - 80) |  |
| **HLA DP** |  |  | 0.801 |
| Non permissive | 14 | 52 (30 - 89) |  |
| Matched/Permissive | 40 | 60 (46 - 78) |  |
| **Conditioning regimen** |  |  | 0.346 |
| TBF | 37 | 53 (39 - 73) |  |
| FTBI | 11 | 81 (60 - 100) |  |
| FMT | 6 | 50 (22 - 100) |  |
| **CD34^+^ cell count in the graft** |  |  | 0.809 |
| ≤ 3.08 x 10^6^/kg | 26 | 57 (40 - 80) |  |
| > 3.08 x 10^6^/kg | 25 | 55 (37 - 81) |  |
| **T cell count in the graft** |  |  | 0.473 |
| ≤ 3.28 x 10^7^/kg | 27 | 54 (38 - 77) |  |
| > 3.28 x 10^7^/kg | 26 | 63 (47 - 86) |  |
| **Immunosuppression** |  |  | 0.087** |
| Cyclosporine A | 41 | 66 (52 - 83) |  |
| Tacrolimus | 13 | 38 (19 - 76) |  |

P values determined using log-rank test.

* The result is not considered significant because the significant p-value for the OS curve in the recipient-donor relation is due to the Niece and Nephew group. Other curves show no significant differences. With only 4 patients in the Niece and Nephew group, the p-value is not robust and thus not meaningful.

** The results are not considered significant due to differences in group size and confounding variables, including recipient age, HCT-CI score, and DRI, which are associated with the outcome.

**Supplementary Table 5 Variables Not Significantly Associated with Graft-versus-host disease-free, Relapse-free Survival (GRFS)**

|  | **N** | **6-year GRFS estimate (95% CI), %** | **P value** |
| --- | --- | --- | --- |
| **Recipient age groups** |  |  | 0.219 |
| 23 - 55 years | 27 | 52 (35 - 77) |  |
| 56 - 75 years | 27 | 32 (19 - 57) |  |
| **Disease** |  |  | 0.354 |
| Myeloid | 37 | 38 (24 - 58) |  |
| Non-myeloid | 17 | 50 (31 - 83) |  |
| **HCT-CI score** |  |  | 0.369 |
| Low | 21 | 57 (39 - 83) |  |
| Intermediate | 16 | 40 (21 - 75) |  |
| High | 17 | 26 (11 - 62) |  |
| **DRI risk categories** |  |  | 0.125 |
| Low - intermediate | 33 | 52 (37 - 73) |  |
| High - very high | 21 | 27 (13 - 56) |  |
| **Donor gender** |  |  | 0.293 |
| Male | 35 | 47 (33 - 68) |  |
| Female | 19 | 31 (15 - 65) |  |
| **Recipient-donor relation** |  |  | 0.485 |
| Father/mother | 8 | 38 (15 - 92) |  |
| Sister/brother | 17 | 57 (37 - 88) |  |
| Son/daughter | 25 | 42 (26 - 67) |  |
| Niece/nephew | 4 | 0 (NA - NA) |  |
| **Donor age** |  |  | 0.647 |
| 19 - 37 years | 26 | 44 (27 - 72) |  |
| 38 - 74 years | 28 | 39 (25 - 62) |  |
| **CMV recipient-donor status** |  |  | 0.903 |
| Negative-negative | 9 | 42 (18 - 94) |  |
| Negative-positive | 9 | 56 (31 - 100) |  |
| Positive-negative | 5 | 20 (3 - 100) |  |
| Positive-positive | 31 | 43 (28 - 65) |  |
| **Donor KIR genotype** |  |  | 0.484 |
| AA | 15 | 32 (15 - 68) |  |
| B+ | 39 | 46 (32- 66) |  |
| **HLA DP** |  |  | 0.634 |
| Non permissive | 14 | 36 (18 - 72) |  |
| Matched/Permissive | 40 | 44 (30 - 64) |  |
| **Conditioning regimen** |  |  | 0.561 |
| TBF | 37 | 43 (29 - 63) |  |
| FTBI | 11 | 36 (17 - 79) |  |
| FMT | 6 | 50 (22 - 100) |  |
| **CD34^+^ cell count in the graft** |  |  | 0.545 |
| ≤ 3.08 x 10^6^/kg | 26 | 33 (18 - 58) |  |
| > 3.08 x 10^6^/kg | 25 | 45 (28 - 71) |  |
| **T cell count in the graft** |  |  | 0.471 |
| ≤ 3.28 x 10^7^/kg | 27 | 39 (24 - 64) |  |
| > 3.28 x 10^7^/kg | 26 | 47 (31 - 72) |  |
| **Immunosuppression** |  |  | 0.198 |
| Cyclosporine A | 41 | 49 (35 - 68) |  |
| Tacrolimus | 13 | 23 (9 - 62) |  |

P values determined using log-rank test.

**Supplementary Table 6 Variables Not Significantly Associated with Cumulative Incidence of Relapse (CIR)**

|  | **N** | **6-year CIR estimate (95% CI), %** | **P value** |
| --- | --- | --- | --- |
| **Gender recipient** |  |  | 0.366 |
| Male | 36 | 24 (13 - 43) |  |
| Female | 18 | 40 (22 - 71) |  |
| **Disease** |  |  | 0.277 |
| Myeloid | 37 | 35 (22 - 55) |  |
| Non-myeloid | 17 | 18 (7 - 51) |  |
| **HCT-CI score** |  |  | 0.599 |
| Low | 21 | 19 (8 - 46) |  |
| Intermediate | 16 | 20 (7 - 55) |  |
| High | 17 | 49 (30 - 81) |  |
| **Donor gender** |  |  | 0.435 |
| Male | 35 | 32 (20 - 52) |  |
| Female | 19 | 25 (11 - 60) |  |
| **Recipient-donor relation** |  |  | 0.13 |
| Father/mother | 8 | 14 (2 - 88) |  |
| Sister/brother | 17 | 19 (7 - 54) |  |
| Son/daughter | 25 | 33 (19 - 58) |  |
| Niece/nephew | 4 | 75 (43 - 100) |  |
| **Donor age** |  |  | 0.914 |
| 19 - 37 years | 26 | 28 (15 - 53) |  |
| 38 - 74 years | 28 | 30 (17 - 54) |  |
| **CMV recipient-donor status** |  |  | 0.384 |
| Negative-negative | 9 | 11 (2 - 71) |  |
| Negative-positive | 9 | 11 (2 - 71) |  |
| Positive-negative | 5 | 40 (14 - 100) |  |
| Positive-positive | 31 | 27 (15 - 49) |  |
| **Donor KIR genotype** |  |  | 0.732 |
| AA | 15 | 34 (17 - 70) |  |
| B+ | 39 | 28 (16 - 47) |  |
| **HLA DP** |  |  | 0.988 |
| Non permissive | 14 | 33 (15 - 73) |  |
| Matched/Permissive | 40 | 29 (17 - 48) |  |
| **Conditioning regimen** |  |  | 0.782 |
| TBF | 37 | 32 (20 - 52) |  |
| FTBI | 11 | 19 (5 - 67) |  |
| FMT | 6 | 33 (11 - 100) |  |
| **CD34^+^ cell count in the graft** |  |  | 0.546 |
| ≤ 3.08 x 10^6^/kg | 26 | 27 (14 - 51) |  |
| > 3.08 x 10^6^/kg | 25 | 37 (21 - 65) |  |
| **T cell count in the graft** |  |  | 0.737 |
| ≤ 3.28 x 10^7^/kg | 27 | 30 (17 - 54) |  |
| > 3.28 x 10^7^/kg | 26 | 29 (15 - 54) |  |
| **Immunosuppression** |  |  | 0.173 |
| Cyclosporine A | 41 | 23 (13 - 41) |  |
| Tacrolimus | 13 | 46 (26 - 83) |  |

P values determined using gray test.

**Supplementary Table 7 Variables Not Significantly Associated with Non-relapse Mortality (NRM)**

|  | **N** | **6-years NRM estimate (95% CI), %** | **P value** |
| --- | --- | --- | --- |
| **Recipient age groups** |  |  | 0.418 |
| 23 - 55 y | 27 | 10 (3 - 37) |  |
| 56 - 75 y | 27 | 15 (6 - 37) |  |
| **Disease** |  |  | 0.397 |
| Myeloid | 37 | 14 (6 - 31) |  |
| Non-myeloid | 17 | 8 (1 - 53) |  |
| **HCT-CI score** |  |  | 0.381 |
| Low | 21 | 5 (1 -32) |  |
| Intermediate | 16 | 23 (8 - 62) |  |
| High | 17 | 12 (3 -43) |  |
| **DRI risk categories** |  |  | 0.697 |
| Low - intermediate | 33 | 14 (5 - 35) |  |
| High - very high | 21 | 10 (3 - 36) |  |
| **Donor gender** |  |  | 0.98 |
| Male | 35 | 12 (5 - 30) |  |
| Female | 19 | 12 (3 - 43) |  |
| **Female donor to male recipient** | |  | 0.485 |
| No | 41 | 11 (4 - 27) |  |
| Yes | 13 | 18 (5 - 64) |  |
| **Recipient-donor relation** |  |  | 0.793 |
| Father/mother | 8 | 14 (2 - 88) |  |
| Sister/brother | 17 | 6 (1 - 39) |  |
| Son/daughter | 25 | 12 (4 - 36) |  |
| Niece/nephew | 4 | 25 (5 - 100) |  |
| **Donor age** |  |  | 0.469 |
| 19 - 37 years | 26 | 10 (3 - 39) |  |
| 38 - 74 years | 28 | 15 (6 - 36) |  |
| **CMV recipient-donor status** |  |  | 0.668 |
| Negative-negative | 9 | 11 (2 - 71) |  |
| Negative-positive | 9 | 0 (NA - NA) |  |
| Positive-negative | 5 | 20 (3 - 100) |  |
| Positive-positive | 31 | 15 (6 - 37) |  |
| **Donor KIR genotype** |  |  | 0.809 |
| AA | 15 | 13 (4 - 48) |  |
| B+ | 39 | 12 (5 -30) |  |
| **HLA DP** |  |  | 0.681 |
| Non permissive | 14 | 15 (4 - 55) |  |
| Matched/Permissive | 40 | 11 (4 - 29) |  |
| **Conditioning regimen** |  |  | 0.415 |
| TBF | 37 | 15 (6 - 33) |  |
| FTBI | 11 | 0 (NA - NA) |  |
| FMT | 6 | 17 (3 - 100) |  |
| **CD34^+^ cell count in the graft** |  |  | 0.534 |
| ≤ 3.08 x 10^6^/kg | 26 | 16 (7 - 40) |  |
| > 3.08 x 10^6^/kg | 25 | 8 (2 - 32) |  |
| **T cell count in the graft** |  |  | 0.474 |
| ≤ 3.28 x 10^7^/kg | 27 | 16 (6 - 40) |  |
| > 3.28 x 10^7^/kg | 26 | 8 (2 - 30) |  |
| **Immunosuppression** |  |  | 0.622 |
| Cyclosporine A | 41 | 11 (4 - 29) |  |
| Tacrolimus | 13 | 15 (4 - 55) |  |

P values determined using gray test.

**Supplementary Table 8 Variables Not Significantly Associated with Acute Graft-versus-Host Disease (GVHD) Grade II-IV**

|  | **N** | **100-day acute GVHD II-IV incidence (95% CI), %** | **P value** |
| --- | --- | --- | --- |
| **Gender recipient** |  |  | 0.397 |
| Male | 36 | 38 (24 - 59) |  |
| Female | 18 | 22 (9 - 53) |  |
| **Recipient age groups** |  |  | 0.572 |
| 23 - 55 years | 27 | 35 (21 - 61) |  |
| 56 - 75 years | 27 | 29 (15 - 55) |  |
| **Disease** |  |  | 0.222 |
| Myeloid | 37 | 27 (15 - 48) |  |
| Non-myeloid | 17 | 43 (24 - 77) |  |
| **HCT-CI score** |  |  | 0.663 |
| Low | 21 | 29 (15 - 57) |  |
| Intermediate | 16 | 28 (12 - 66) |  |
| High | 17 | 42 (22 - 79) |  |
| **DRI risk categories** |  |  | 0.239 |
| Low - intermediate | 33 | 39 (25 - 61) |  |
| High - very high | 21 | 22 (9 - 54) |  |
| **Donor gender** |  |  | 0.783 |
| Male | 35 | 31 (18 - 53) |  |
| Female | 19 | 36 (18 - 69) |  |
| **Female donor to male recipient** | |  | 0.409 |
| No | 41 | 29 (17 - 48) |  |
| Yes | 13 | 47 (24 - 92) |  |
| **Recipient-donor relation** |  |  | 0.133 |
| Father/mother | 8 | 75 (45 - 100) |  |
| Sister/brother | 17 | 30 (15 - 64) |  |
| Son/daughter | 25 | 27 (13 - 54) |  |
| Niece/nephew | 4 |  |  |
| **Donor age** |  |  | 0.088 |
| 19 - 37 years | 26 | 20 (9 - 45) |  |
| 38 - 74 years | 28 | 45 (29 - 71) |  |
| **CMV recipient-donor status** |  |  | 0.854 |
| Negative-negative | 9 | 22 (7 - 75) |  |
| Negative-positive | 9 | 38 (12 - 100) |  |
| Positive-negative, Positive-positive | 36 | 33 (21 - 54) |  |
| **Donor KIR genotype** |  |  | 0.337 |
| AA | 15 | 46 (25 - 85) |  |
| B+ | 39 | 27 (16 - 46) |  |
| **HLA DP** |  |  | 0.317 |
| Non permissive | 14 | 44 (24 - 80) |  |
| Matched/Permissive | 40 | 28 (16 - 48) |  |
| **CD34^+^ cell count in the graft** |  |  | 0.305 |
| ≤ 3.08 x 10^6^/kg | 26 | 45 (28 - 72) |  |
| > 3.08 x 10^6^/kg | 25 | 24 (12 - 49) |  |
| **T cell count in the graft** |  |  | 0.209 |
| ≤ 3.28 x 10^7^/kg | 27 | 46 (28 - 75) |  |
| > 3.28 x 10^7^/kg | 26 | 25 (11 - 55) |  |
| **Immunosuppression** |  |  | 0.523 |
| Cyclosporine A | 41 | 34 (22 - 54) |  |
| Tacrolimus | 13 | 26 (10 - 69) |  |

P values determined using gray test.

**Supplementary Table 9 Virus Reactivation and Infections**

|  | No data | Entire cohort  (N=54) | No inhibitory KIR/HLA mismatch (N=26) | Inhibitory KIR/HLA mismatch (N=28) | *P* value |
| --- | --- | --- | --- | --- | --- |
| CMV | 7 | 34 (72) | 15 (68) (4*) | 19 (76) (3*) |  |
| BK virus |  | 4 | 0 | 4 |  |
| Adenovirus |  | 2 | 1 | 1 |  |
| Rotavirus |  | 1 | 0 | 1 |  |
| Norovirus |  | 2 | 1 | 1 |  |
| Enterovirus | 1 | 1 | 0 | 1* |  |
| Influenza |  | 3 | 2 | 1 |  |
| RSV |  | 3 | 1 | 2 |  |
| Other respiratory virus | 1 | 9 | 5 | 4* |  |
| Parvovirus B19 | 1 | 0 | 0 | 0* |  |
| EBV | 1 | 7 | 2 | 5* |  |
| Varicella zoster virus |  | 0 | 0 | 0 |  |
| HSV |  | 6 | 3 | 3 |  |
| HHV6 |  | 9 | 4 | 5 |  |
| HBV |  | 1 | 1 | 0 |  |
| Recurrence of different viruses |  |  |  |  |  |
| No virus |  | 12 | 7 | 5 |  |
| Once |  | 27 | 13 | 14 |  |
| Twice |  | 9 | 4 | 5 |  |
| ≥ Trice |  | 6 | 2 | 4 |  |
